# Phosphoinositide-binding activity of Smad2 is essential for its function in TGF-β signaling

**DOI:** 10.1016/j.jbc.2021.101303

**Published:** 2021-10-14

**Authors:** Pawanthi Buwaneka, Arthur Ralko, Sukhamoy Gorai, Ha Pham, Wonhwa Cho

**Affiliations:** Department of Chemistry, University of Illinois at Chicago, Chicago, Illinois, USA

**Keywords:** Smad2, TGF-b, PI(4,5)P__2__, membrane-protein interaction, plasma membrane targeting, TGF-b receptor, MH2 domain, EEs, early endosomes, EGFP, enhanced green fluorescence protein, FKBP12, 12 kDa FK506-binding protein, FRB, FKBP12-rapamycin binding domain of mTOR, GAPDH, glyceraldehyde 3-phosphate dehydrogenase, LUVs, large unilamellar vesicles, MH, Mad homology, PH, pleckstrin homology domain, PI(3)P, phosphatidylinositol-3-phosphate, PI(3,4)P_2_, phosphatidylinositol-3,4-bisphosphate, PI(4,5)P_2_, phosphatidylinositol-4,5-bisphosphate, PIP_3_, phosphatidylinositol-3,4,5-bisphosphate, PLCδ, phospholipase Cδ, PM, plasma membrane, POPC, 1-palmitoyl-2-oleoyl-sn-glycero-3-phosphocholine, POPS, 1-palmitoyl-2-oleoyl-sn-glycero-3-phosphoserine, PtdInsP, phosphoinositide, SARA, Smad anchor for receptor activation, siRNA, short interference RNA, SPR, surface plasmon resonance, TβR, transforming growth factor-β receptor, TGF-β, transforming growth factor-β, TIRF, total internal reflection fluorescence, TMR, tetramethylrhodamine, WT, wild type

## Abstract

As a central player in the canonical TGF-β signaling pathway, Smad2 transmits the activation of TGF-β receptors at the plasma membrane (PM) to transcriptional regulation in the nucleus. Although it has been well established that binding of TGF-β to its receptors leads to the recruitment and activation of Smad2, the spatiotemporal mechanism by which Smad2 is recruited to the activated TGF-β receptor complex and activated is not fully understood. Here we show that Smad2 selectively and tightly binds phosphatidylinositol-4,5-bisphosphate (PI(4,5)P_2_) in the PM. The PI(4,5)P_2_-binding site is located in the MH2 domain that is involved in interaction with the TGF-β receptor I that transduces TGF-β-receptor binding to downstream signaling proteins. Quantitative optical imaging analyses show that PM recruitment of Smad2 is triggered by its interaction with PI(4,5)P_2_ that is locally enriched near the activated TGF-β receptor complex, leading to its binding to the TGF-β receptor I. The PI(4,5)P_2_-binding activity of Smad2 is essential for the TGF-β-stimulated phosphorylation, nuclear transport, and transcriptional activity of Smad2. Structural comparison of all Smad MH2 domains suggests that membrane lipids may also interact with other Smad proteins and regulate their function in diverse TGF-β-mediated biological processes.

In the consensus model of canonical Smad-mediated TGF-β signaling, TGF-β binds to the type II TGF-β receptor (TβRII), which leads to recruitment of the type I TGF-β receptor (TβRI) into a heteromeric receptor complex, enabling TβRII to trans-phosphorylate serines and a threonine in the juxtamembrane Gly-Ser-rich sequence (GS domain) of TβRI (1, 2). The phosphorylation and resulting conformational changes result in the release of FKBP12 and the recruitment of regulatory Smad (R- Smad), most notably Smad2 and Smad3, to the activated TβRI (3). Phosphorylation of TβRI also promotes the dissociation of inhibitory Smads from TβRI (4). TβRI then phosphorylates two C-terminal serines (S465 and S467) of the bound Smad2 (or Smad3), and these receptor-activated R-Smads dissociate from the complex and associate with a co-Smad (Smad4) (1, 2). The Smad2/3/4 complex then enters the nucleus where they associate with other transcription cofactors at Smad-binding regulatory DNA sequences of target genes, thus directly modulating target gene expression (1, 5).

Although this model is widely accepted, there is a major gap in our understanding of the spatiotemporal dynamics of the active TGF-β receptor complex and the mechanism by which Smad2 and/or Smad3 are recruited to the complex. It has been reported that Smad-dependent canonical TGF-β signaling takes place in endocytic clathrin-coated pits of the plasma membrane (PM) (6). It has been also reported that an FYVE domain-containing protein, Smad anchor for receptor activation (SARA), recruits Smad2 and/or Smad3 to the TGF-β receptor complex at early endosomes (EEs) through its binding to phosphatidylinositol-3-monophosphate (PI(3)P) and the endocytosed TGF-β receptor complex at EEs (7). Although these findings suggest that clathrin-dependent internalization of the TGF-β receptors is a crucial step for Smad-mediated TGF-β signaling and that EEs are a main site for Smad-mediated TGF-β signaling, these notions have been challenged by other reports. First, it has been reported that Smad-mediated TGF-β signaling can be initiated from the PM without requirement of endocytosis (8, 9, 10). Furthermore, the direct involvement of SARA in TGF-β signaling has been disputed (11, 12, 13).

In this study, we explored the possibility that targeting of Smad2 to the activated TGF-β receptor complex is controlled by their direct interaction with membrane lipids. Our membrane binding studies as well as quantitative cell imaging studies show that Smad2 binds phosphatidylinositol-4,5-bisphosphate (PI(4,5)P_2_) enriched in the PM more tightly than PI(3)P abundant in EEs. Our results also show that the PI(4,5)P_2_-binding activity of Smad2 is essential for its functional interaction with the activated TGF-β receptor complex at the PM and the overall Smad-dependent TGF-β signaling activity.

## Results

### Smad2 binds PI(4,5)P_2_ with high affinity and specificity

It has been generally thought that Smad2 and Smad3 are targeted to the activated TGF-β signaling complex by direct interaction with TβRI (1, 2) or *via* SARA (7). However, it has been shown that many cytosolic proteins that are targeted to membrane proteins or membrane-anchored signaling complexes have affinity for membrane lipids (14), phosphoinositides (PtdInsPs) in particular, and that their membrane recruitment is mediated by coincident lipid–protein and protein–protein interactions (15, 16). For most of these proteins, their PtdInsP specificity directs their membrane targeting behaviors: *e.g.*, PI(4,5)P_2_-specific proteins are recruited to PM where the highest concentration of PI(4,5)P_2_ is found, whereas PI(3)P-specific proteins are targeted to PI(3)P-rich EEs (17, 18). To resolve uncertainty about the functional location of the activated TGF-β signaling complex, we thus explored the possibility that lipid-binding activity of Smad2 may play a role in specific targeting of Smad2 to the active TGF-β receptor complex either at the PM or at EEs. Lipid-binding activity of Smad proteins has not been reported to date. We thus measured the binding of bacterially expressed Smad2 to large unilamellar vesicles (LUVs) containing various lipids by surface plasmon resonance (SPR) analysis (19, 20). Lipid selectivity determined by SPR analysis is typically reported as the relative resonance unit (RU) values for different lipids at a given protein concentration (21, 22). Although simple and intuitive, this type of analysis can sometimes yield misleading and erroneous results because some proteins show widely different RU values when bound to different lipid surfaces. Thus, a more reliable parameter that represents the fraction of the membrane bound protein molecules at a given protein concentration would be a normalized value of RU/RU_max_ where RU_max_ indicates the maximal RU value when a given lipid surface is saturated with the protein molecules. We thus estimated RU_max_ for different lipid species by employing the highest protein concentration experimentally feasible (*e.g.*, 1 μM) and determined RU/RU_max_ for different lipids at the protein concentration that allows robust comparison (*e.g.*, 100 nM). Once the lipid selectivity has been determined by this semiquantitative approach, we then rigorously determined and compared *K*_d_ values for selected lipids for more accurate quantitative determination of lipid selectivity.

**Figure 1:**
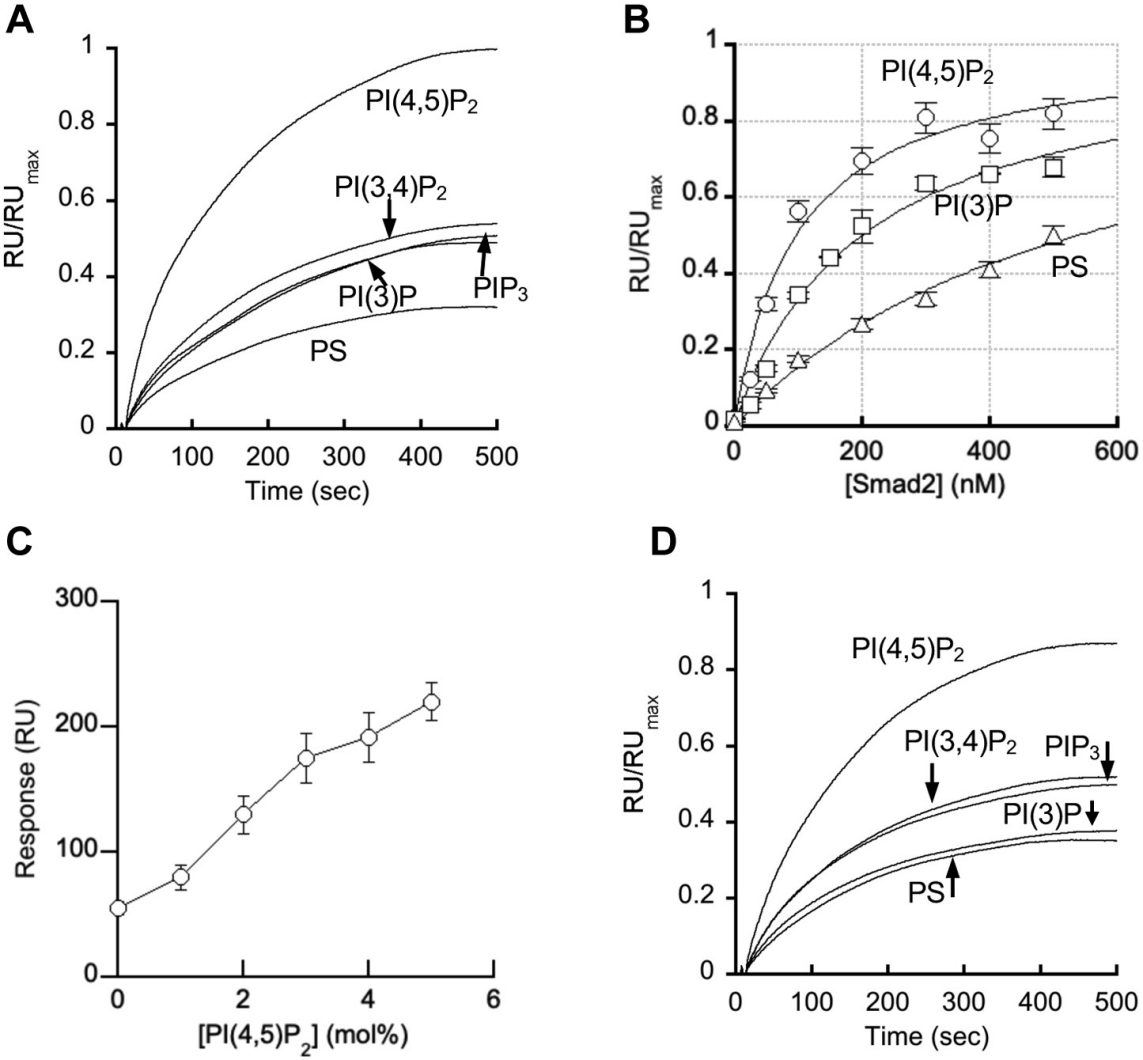
**Membrane-binding properties of Smad2 FL protein and MH2 domain determined by SPR analysis.***A*, PtdInsP selectivity of Smad2-FL determined using POPC/POPS/PtdInsP (77:20:3 in mole ratio) LUVs coated on the L1 sensor chip. The protein concentration was 100 nM. PIP_3_ indicates PI(3,4,5)P_3_. *B*, determination of *K*_d_ for binding of Smad2 FL to different LUVs. The protein concentration was varied from 0 to 500 nM. *C*, PI(4,5)P_2_ concentration dependence of membrane binding of Smad2-FL determined using POPC/POPS/PI(4,5)P_2_ (77-*x*:20:*x*, *x* = 0–5 mol%) LUVs. The protein concentration was 100 nM. *D*, PtdInsP selectivity of the Smad2 MH2 domain determined using POPC/POPS/PtdInsP (77:20:3 in mole ratio) LUVs. The protein concentration was 100 nM. Normalized RU values (RU/RUmax) were used for all figures but (*C*) for more accurate determination of PtdInsP selectivity. RUmax values determined for different vesicles at 1 μM Smad2 were: 280 ± 20 for POPC/POPS/PI(4,5)P_2_ (77:20:3), 370 ± 10 for POPC/POPS/PI(3,4)P_2_ (77:20:3), 400 ± 15 for POPC/POPS/PI(3,4,5)P_3_ (or PIP_3_) (77:20:3), 400 ± 20 for POPC/POPS/PI(3)P (77:20:3), and 380 ± 20 for POPC/POPS (80:20). Only the association phases of the sensorgrams are shown and used for further analysis. Each set of sensorgrams shown in *A* and *D* are representatives of >5 independent measurements. Data points shown in *B* and *C* are the average ±S.D. values from triplicate measurements.

We first determined by the SPR analysis the PtdInsP selectivity of Smad2 using 1-Palmitoyl-2-oleoyl-sn-glycero-3-phosphocholine (POPC)/1-palmitoyl-2-oleoyl-sn-glycero-3-phosphoserine (POPS)/PtdInsP (77:20:3 in molar ratio) LUVs coated onto the L1 sensor chip, with a focus on its relative binding affinity for PI(4,5)P_2_ *versus* PI(3)P. As shown in Figure 1*A*, Smad2 binds PtdInsP-containing anionic vesicles better than POPC/POPS (80:20) vesicles and has a significant degree of selectivity for POPC/POPS/PI(4,5)P_2_ (77:20:3) over other PtdInsP-containing vesicles, including POPC/POPS/PI(3)P (77:20:3). The fact that Smad2 can distinguish between two similar bisphosphates, PI(4,5)P_2_ and phosphatidylinositol-3,4-bisphosphate (PI(3,4)P_2_), indicates that it has a PI(4,5)P_2_-specific binding pocket. To quantitatively assess its membrane affinity, we then determined the *K*_d_ values for selected vesicles by measuring RU/RU_max_ as a function of the Smad2 concentration (Fig. 1*B*). The *K*_d_ values (Table 1) confirmed that Smad2 has twofold higher affinity for PI(4,5)P_2_-containing vesicles than for PI(3)P-containing ones and that 3 mol% PI(4,5)P_2_ caused a large 5.6-fold increase in its membrane affinity over POPC/POPS (80:20) vesicles. We further investigated the PI(4,5)P_2_ dependence of its membrane binding by measuring RU as a function of PI(4,5)P_2_ concentration in the vesicles (*i.e.*, POPC/POPS/PI(4,5)P_2_ (77-*x*:20:*x*, *x* = 0–5 mol%)) (Fig. 1*C*). The result verifies that membrane binding of Smad2 is dependent on the PI(4,5)P_2_ concentration. Taken together, our SPR data show that Smad2 is a PI(4,5)P_2_-dependent membrane-binding protein. They also suggest that its high affinity and selectivity for PI(4,5)P_2_-containing membranes may dictate its PM recruitment and targeting to the activated TGF-β signaling complex at the PM.

**Table 1:** Lipid and peptide-binding activity of Smad2 proteins

Proteins	*K*_d_ for lipid binding (nM)^a^			*K*_d_ for peptide binding (μM)^b^	
	PC/PS/PI(4,5)P_2_	PC/PS/PI(3)P	PC/PS	GS peptide	L45 peptide
Smad2 WT	100 ± 20	210 ± 34	560 ± 150	20 ± 6	66 ± 4
Smad2-K420A	120 ± 12	ND^c^	ND	200 ± 48	100 ± 16
Smad2-W422A	470 ± 96	ND	ND	21 ± 5	67 ± 6
Smad2-E425K	70 ± 7	ND	ND	8 ± 0.8	55 ± 3
Smad2-Y426A	400 ± 44	ND	ND	20 ± 3	70 ± 7
Smad2- R427A/R428A	340 ± 53	ND	ND	44 ± 4	75 ± 11
Smad3 WT	170 ± 15	ND	ND	ND	ND

a Average ±S.D. values determined from SPR analysis (*n* = 3); see Figure 1*B* for experimental details.

b Average ±S.D. values determined from fluorescence anisotropy analysis (*n* = 3); See Figure 3, *A* and *B* for experimental details.

c Not determined.

### The MH2 domain contains the PI45P_2_-binding site

**Figure 2:**
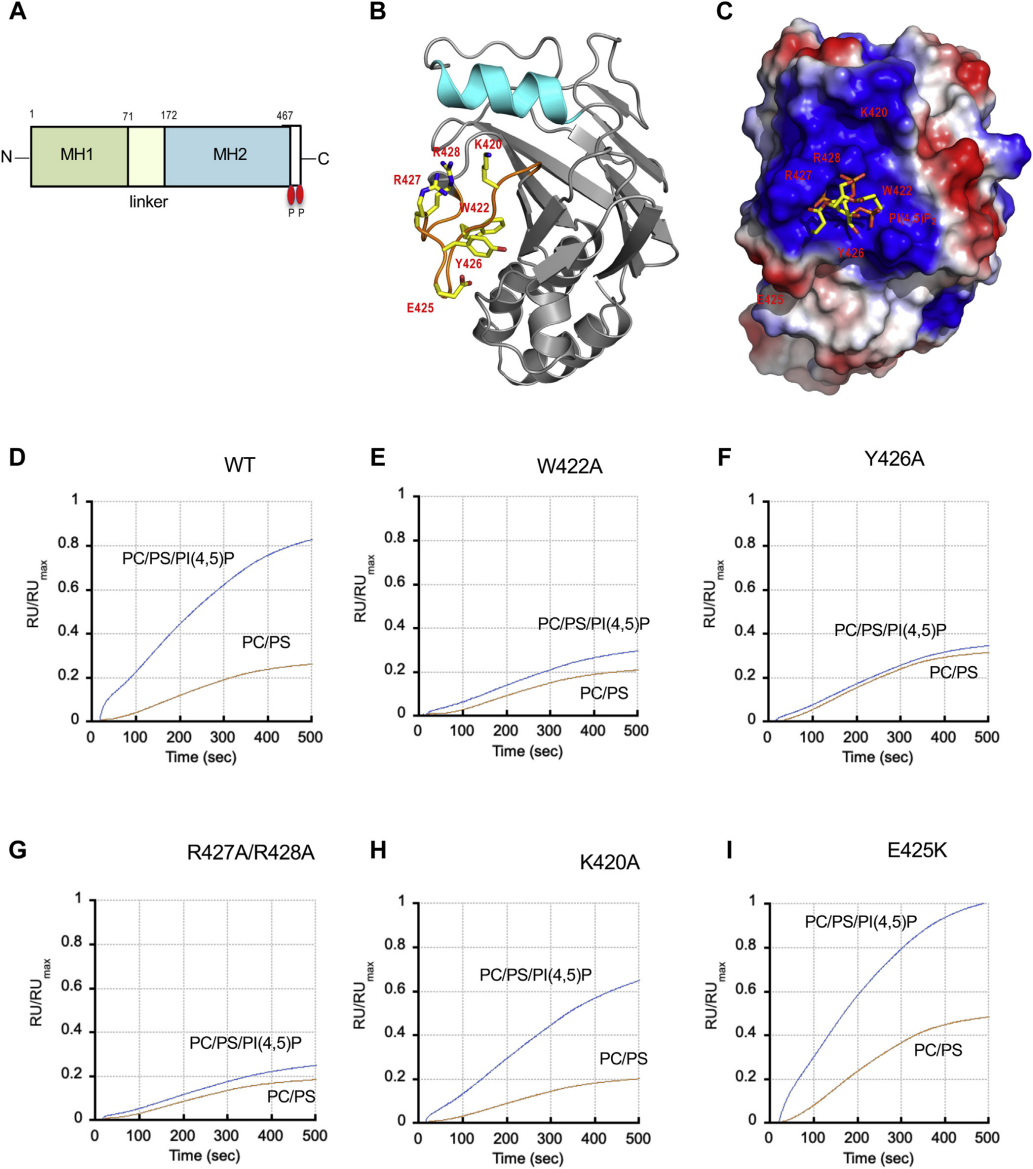
**Identification of the membrane-binding site of Smad2 by the structure–function analysis.***A*, schematic representation of the Smad2 domain structure. The MH1 and MH2 domains are connected by a flexible linker. The C-terminal SMS motif that is phosphorylated by TβRI is indicated by *red ovals*. *B*, the structure of the Smad2 MH2 domain (protein data bank ID: 1DEV) shown in a *ribbon* diagram. The H3 helix and L3 loop are highlighted in *cyan* and *orange*, respectively. Those residues involved in membrane binding are shown in *stick* representation and labeled. The molecule is oriented with its membrane-binding surface facing the viewer. *C*, the electrostatic potential map (generated by Pymol) of the same structure in surface representation with a PI(4,5)P_2_ molecule (*stick* representation) docked into the cationic pocket. *Blue* and *red* indicate positive and negative electrostatic potentials. *D*–*I*, selectivity of Smad2 WT (*D*), W422A (*E*), Y420A (*F*), R427A/R428A (*G*), K420A (*H*), and E425K (*I*) for POPC/POPS/PI(4,5)P_2_ (77:20:3) (*blue*) over POPC/POPS (80:20) (*orange*) vesicles determined by SPR analysis. The protein concentration was 100 nM. Notice that W422A, Y426A, and R427A/R428A show essentially no PI(4,5)P_2_ selectivity. RU/RUmax values were calculated as described for Figure 1. Only the association phases of the sensorgrams are shown and used for further analysis. Each set of sensorgrams shown in *D*–*I* are representatives of three independent measurements.

Smad2 has two conserved domains, the N-terminal MH1 and C-terminal MH2 domain, connected by a variable linker region (Fig. 2*A*) (1, 2, 23). The MH1 domain is involved in nuclear localization of Smad2, whereas the MH2 domain in interaction with the activated TβRI and SARA (1, 2, 23, 24). Lipid-binding sites of cytosolic proteins typically contain a cluster of cationic and aromatic residues (15). Electrostatic potential calculation and surface cavity analysis (21, 25, 26, 27) of a reported crystal structure of Smad2 (24) identified a wide and shallow cationic groove containing aromatic residues in the MH2 domain (Fig. 2, *B* and *C*). Molecular docking analysis using the Smad2 structure and a short-chain PI(4,5)P_2_ model also suggested that the pocket could accommodate a PI(4,5)P_2_ headgroup making energetically favorable interactions with the 4′- and 5′-phosphate groups of the PI(4,5)P_2_ molecule (Fig. 2*C*). When we expressed the isolated MH2 domain and measured its membrane binding by SPR analysis, it had similar PtdInsP selectivity to the full-length (FL) Smad2 (Fig. 1*D*), supporting the notion that the MH2 domain contains the PI(4,5)P_2_-binding site.

PtdInsP binding of cytosolic proteins is typically driven by two types of interactions, nonspecific contact between the protein and membrane surfaces and specific recognition of a PtdInsP molecule in the binding pocket of the protein (15). To identify protein residues involved in the two types of interactions, we performed a series of single- or double-site mutations of residues within (*i.e.*, W422, Y426, R427, and R428) and surrounding (*i.e.*, K420) the putative PI(4,5)P_2_-binding pocket and measured the effects of the mutations on binding to two different vesicles, POPC/POPS/PI(4,5)P_2_ (77:20:3) and POPC/POPS (80:20). The former vesicles are for evaluating specific PI(4,5)P_2_ recognition and the latter for nonspecific binding to anionic membranes. Because of relatively low stability of the MH2 domain, we performed membrane-binding measurements using the FL Smad2 wild-type (WT) (Fig. 2*D*) and mutants (Fig. 2, *E*–*G*). All mutants were expressed stably and in good yield in *E. coli*, indicating that the mutations did not cause deleterious gross conformational changes. As shown in Figure 2, *E*–*G*, W422A, Y426A, and R427A/R428A mutations greatly reduced binding to POPC/POPS/PI(4,5)P_2_ (77:20:3) while showing much less effects on binding to POPC/POPS (80:20). Consequently, these mutants lost the PI(4,5)P_2_ selectivity of the WT Smad2 (Fig. 2*D*). In contrast, the K420A mutation modestly and similarly reduced binding to both vesicles (Fig. 2*H*). These results thus indicate that W422, Y426, R427, and R428 are involved in specific PI(4,5)P_2_ binding, whereas K420 participates in nonspecific electrostatic interaction with the anionic membrane surface. This notion is consistent with our model predicting that W422, Y426, R427, and R428 constitutes the cationic groove, whereas K420 is located on the protein surface flanking the groove (Fig. 2*C*). Interestingly, an anionic residue, E425, is located on the same membrane-contacting surface as K420, presumably interfering with nonspecific electrostatic interaction with the anionic membrane surface (Fig. 2, *B* and *C*). We thus prepared a charge-reversal mutant (E425K) to generate a potential gain-of-function mutant. As shown in Figure 2*I*, E425K showed modestly higher membrane binding than WT while retaining the PI(4,5)P_2_ selectivity of WT. Lastly, we determined the *K*_d_ values for these Smad2 mutants. As summarized in Table 1, W422A, Y426A, and R427A/R428A have three- to five-fold lower affinity for POPC/POPS/PI(4,5)P_2_ (77:20:3) than WT. On the other hand, E425K has ≂40% higher affinity than WT, whereas K420A has only 20% lower affinity than WT.

**Figure 3:**
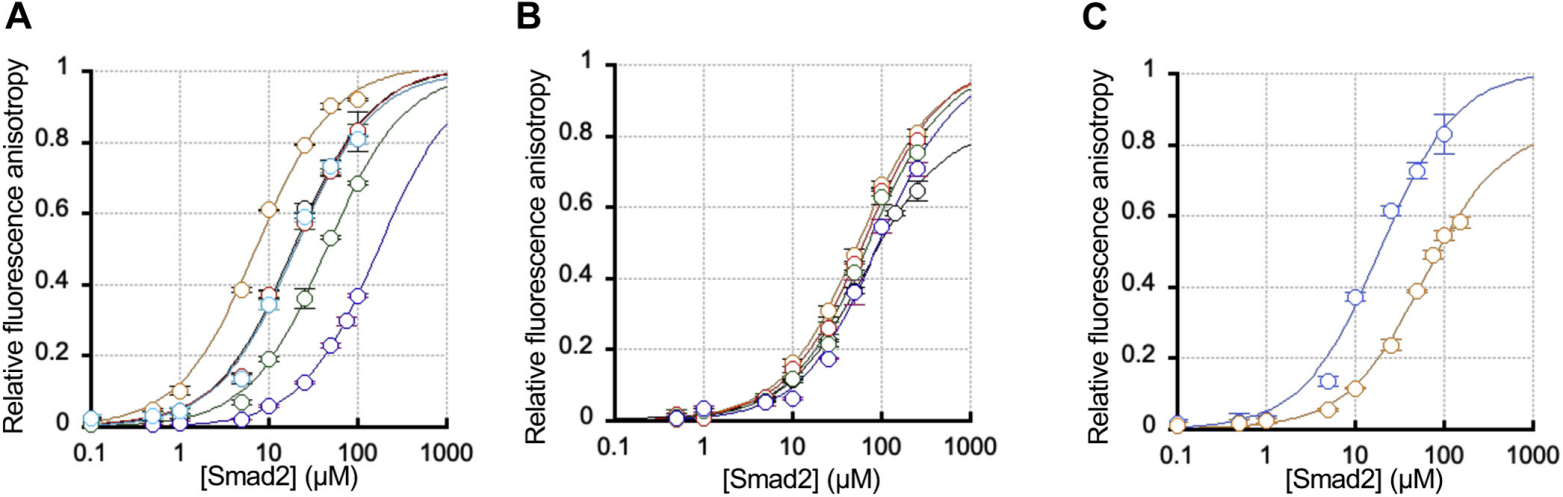
**Affinity of Smad2 WT and mutants for TβRI-derived peptides determined by fluorescence anisotropy.***A*, binding of Smad2 WT (*black*), W422A (*red*), Y420A (*cyan*), R427A/R428A (*green*), K420A (*blue*), and E425K (*orange*) to the GS peptide (F-Ahx-YDMTTpSGpSGpSGLPLL). WT, W422A, and Y420A showed essentially identical binding curves. *B*, binding of Smad2 WT (*black*), W422A (*red*), Y420A (*cyan*), R427A/R428A (*green*), K420A (*blue*), and E425K (*orange*) to the L45 loop peptide (F-Ahx-DNKDNGT). *C*, effects of 50 μM POPC/POPS/PI(4,5)P_2_ (77:20:3) LUVs on binding of Smad2 to the GS peptide. The presence of vesicles (*orange*) raises the *K*_d_ value from 20 ± 6 to 60 ± 7 nM. The peptide concentration was fixed at 2.5 nM, and the Smad2 concentration was varied from 0 to 150 μM. The experimentally observed anisotropy (*A*) values were normalized (*A*_norm_) using the equation: *A*_norm_ = (*A* − *A*_min_)/(*A*_max_ − *A*_min_) where *A*_max_ and *A*_min_ are maximal and minimal *A* values, respectively, for each measurement. The plots of *A*_norm_*versus* [Smad2] were analyzed by the nonlinear least-squares analysis using the equation: *A*_norm_ = 1/(1 + *K*_d_/[Smad2]).

Earlier mutational studies (3, 28, 29) suggested that two regions of the Smad2 MH2 domain, the H1 helix and L3 loop (see Fig. 2*B*), may be involved in interaction with the GS domain (aa 185–194) and the L45 loop (aa 265–273) of TβRI, respectively. However, this tentative assignment has been confirmed neither by a high-resolution structure of the Smad2-TβRI complex nor by a quantitative and systematic binding study, leaving uncertainty about the exact nature of the Smad2-TβRI-binding interface. Since the L3 loop is a part of the newly identified PI(4,5)P_2_-binding site, we checked if these PI(4,5)P_2_-binding residues are also involved in the Smad2-TβRI interaction using two fluorescein-labeled synthetic peptides derived from the GS region (*i.e.*, GS peptide: fluorescein-aminohexanoyl (F-Ahx)-YDMTT**pS**G**pS**G**pS**GLPLL) and the L45 loop (*i.e.*, L45 peptide: F-Ahx-ADNKDNGT), respectively. In particular, the GS peptide represents the GS motif triply phosphorylated by TβRII. We then measured binding to Smad2 WT and mutants to these peptides by fluorescence anisotropy according to our established protocol (21). As shown in Figure 3, *A* and *B* (see also Table 1), Smad2 bound the GS peptide 3.3 times more tightly than the L45 peptide, indicating that the phosphorylated GS domain plays a more important role in Smad2 binding than the L45 region. We thus primarily used the GS peptide to assess the effects of mutations on the Smad2-TβRI interaction. As shown in Figure 3*A* (see also Table 1), the K420A mutation caused a large tenfold decrease in Smad2-GS peptide binding, indicating that K420 is directly involved in the Smad2-TβRI interaction. R427A/R428A have twofold lower affinity for the GS peptide than the Smad2 WT, whereas E425K has twofold higher affinity, suggesting the partial involvement of these residues in the peptide binding. In contrast, W422A and Y426A have essentially the same affinity as the WT, showing that these residues, which play a critical role in PI(4,5)P_2_ binding, are not involved in the peptide binding. Although Smad2 has lower affinity for the L25 peptide than for the GS peptide, mutants show similar trends: *i.e.*, K420A with the lowest affinity, R427A/R428A with slightly lower affinity, E425K with slightly higher affinity, and W422A and Y426A with WT-like affinity (Fig. 3*B* and Table 1). Taken together, these results suggest that those residues in the PI(4,5)P_2_-binding pocket (*e.g.*, W422 and Y426) of Smad2 are not involved in TβRI binding, whereas those residues on the nonspecific membrane-binding surface (*e.g.*, K420) are more directly involved in TβRI binding. They also yielded distinct structural variants for further functional studies. Specifically, W422A and Y426A could serve as specific PI(4,5)P_2_ binding-compromised mutants and K420A as a predominantly TβRI binding-compromised mutant. R427A/R428A could also serve as a PI(4,5)P_2_ and TβRI binding-compromised mutant, whereas E425K as a PI(4,5)P_2_ and TβRI binding-enhanced mutant.

To check if a partial overlap between the lipid-binding and TβRI-binding interfaces makes the two binding processes mutually exclusive, we performed the peptide-binding measurement in the presence of LUVs. As shown in Figure 3*C*, the presence of 50 μM POPC/POPS/PI(4,5)P_2_ (77:20:3) LUVs in the reaction mixture significantly interfered with binding of Smad2 to the GS peptide when assayed by fluorescence anisotropy. This result suggests that although the overlap between the lipid and protein binding sites in the MH2 domain of Smad2 is relatively modest, this partial overlap may not allow coincident binding of Smad2 to PI(4,5)P_2_ in the PM and the juxtamembrane TβRI region. It is therefore more likely that Smad2 interacts with membrane lipids and TβRI sequentially.

### The cellular PI(4,5)P_2_-binding activity of Smad2 is essential for its PM recruitment and interaction with TβRI

**Figure 4:**
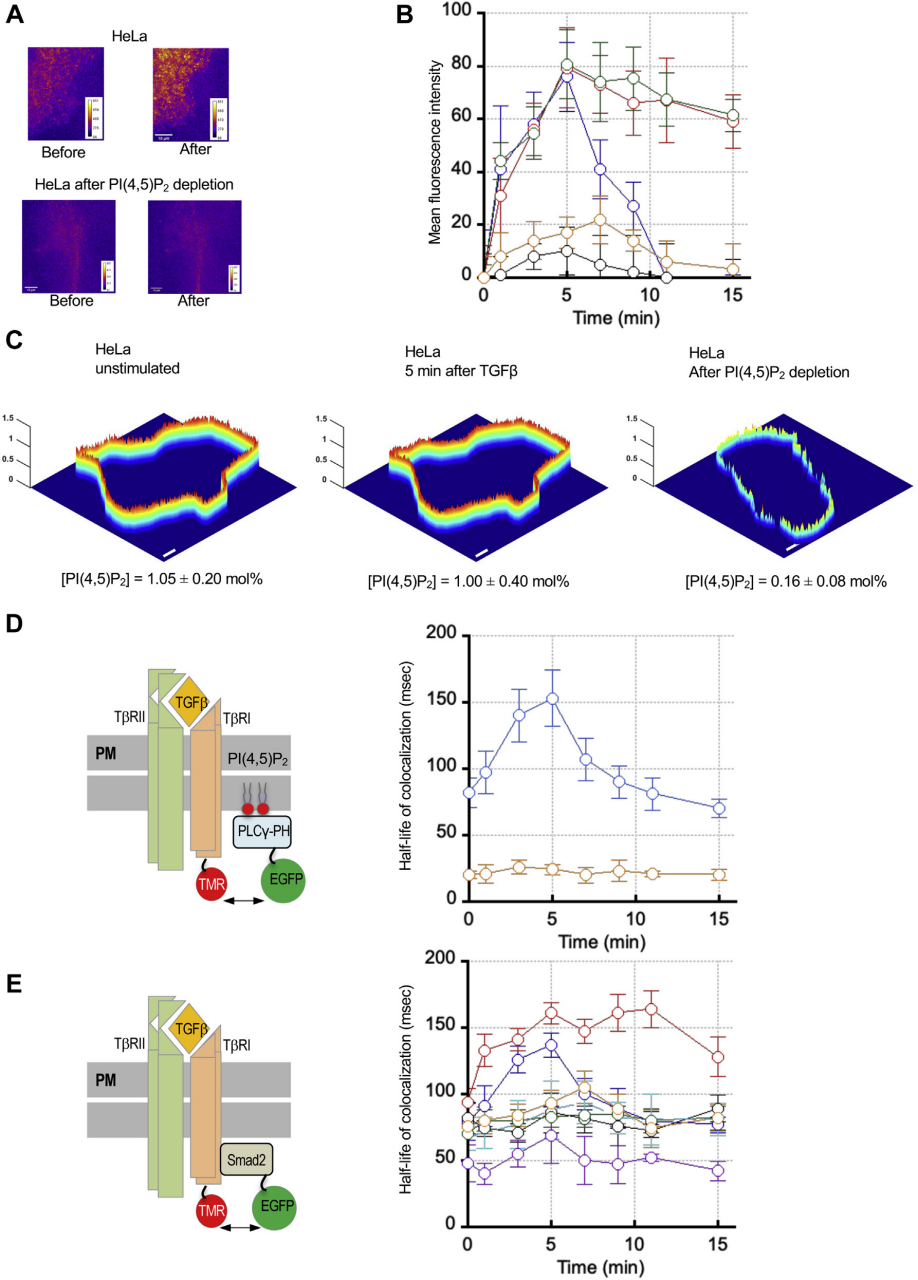
**Quantitative imaging analyses of spatiotemporal dynamics of Smad2.***A*, TIRF images of mouse EGFP-Smad2-WT transfected into HeLa cells whose endogenous Smad2 is suppressed by siRNA. Images were taken before and 5 min after TGF-β stimulation (10 ng/ml) with and without PI(4,5)P_2_ depletion. A pseudo-coloring scheme was used with *orange* and *blue* representing the highest and the lowest intensity, respectively. *B*, kinetics of TGF-β stimulated (10 ng/ml) PM localization of EGFP-Smad2-WT (*blue*), -K420A (*green*), and -W422A (*orange*) transfected into HeLa cells the endogenous Smad2 of which was suppressed by siRNA. For EGFP-Smad2-WT, the effect of PI(4,5)P_2_ depletion (*black*) and TβRI inhibition by SB-431542 (10 μM for 36 h) (*red*) were measured. Data points indicate average ±S.D. (*n* = 10 cells). *C*, spatially resolved PM PI(4,5)P_2_ concentration ([PI(4,5)P_2_]) profiles calculated from two-channel cross-sectional images of HeLa cells (before and 5 min after 10 ng/ml TGF-β stimulation and after PI(4,5)P_2_ depletion). Each cell is a representative of more than ten cells analyzed. Spatially averaged [PI(4,5)P_2_] values for each cell type are shown. The *z*-axis scale indicates [PI(4,5)P_2_] in mol%. A pseudo-coloring scheme with *red* and *blue* representing the highest (1.5 mol%) and the lowest (0 mol%) concentration, respectively, is used to illustrate the spatial [PI(4,5)P_2_] heterogeneity. Scale bars indicate 10 μm. *D*, dual color single-molecule tracking of EGFP-PLCδ-PH and SNAP-TMR-labeled TβRI at the PM of HeLa cells. Time courses of the half-life of colocalization for SNAP-TMR-labeled TβRI and EGFP-PLCδ-PH after 10 ng/ml TGF-β stimulation were determined with (*orange*) and without (*blue*) PI(4,5)P_2_ depletion. Data points indicate average ±S.D. (*n* = 10 cells). *E*, time courses of the half-life of colocalization for SNAP-TMR-labeled TβRI and EGFP-Smad2 determined by dual color single-molecule tracking analysis. Colocalization of TβRI with EGFP-Smad2-WT (*blue*), -K420A (*green*), -W422A (*orange*), E425K (*red*), Y426A (*cyan*), and -R427A/R428A (*black*) transfected into HeLa cells whose endogenous Smad2 was suppressed by siRNA is shown. For EGFP-Smad2-WT, the effect of PI(4,5)P_2_ depletion (*purple*) was measured. Data points indicate average ±S.D. (*n* = 10 cells). For *B* and *E*, only those cells with comparable protein expression levels were selected for image analysis.

Membrane translocation of Smad2 in response to TGF-β stimulation has not been demonstrated presumably due to the transient nature of its membrane residence. It has been reported that Smad2 is mostly located in the cytoplasm of unstimulated mammalian cells (30, 31, 32). We also found that in unstimulated HeLa cells, endogenous Smad2 or exogenously expressed EGFP-Smad2 mostly distributed in the cytoplasm with little prelocalization to the PM (Fig. S1*A*). Even after TGF-β stimulation, it was still difficult to clearly visualize PM (or EE) localization of endogenous Smad2 or exogenously expressed EGFP-Smad2 by confocal microscopy (Fig. S1*B*). We thus employed the total internal reflection fluorescence (TIRF) microscopy (33) that allows more sensitive and quantitative detection of PM translocation of cytosolic proteins. When monitored by the TIRF microscopy, TGF-β-stimulated PM recruitment of EGFP-Smad2 transfected into HeLa cells was clearly visible (Fig. 4*A*). Quantification of fluorescence intensity signals showed that PM translocation of Smad2 WT peaked at 5 min after TGF-β stimulation and declined rapidly afterward (Fig. 4*B*). This PM translocation was driven by PI(4,5)P_2_ binding of Smad2 because it was abrogated (Fig. 4, *A* and *B*) when PI(4,5)P_2_ at the PM was depleted (Fig. 4*C*) by a yeast inositol polyphosphate 5-phosphatase, lnp54, whose PM translocation is triggered by rapamycin-induced dimerization of Lyn-FKBP12 and FRB-Inp54 (34, 35). Also, rapid dissociation of Smad2 from the PM was due to TβRI-catalyzed phosphorylation of Smad2 because inhibition of TβRI kinase activity by SB-431542 greatly elongated the PM residence of Smad2 (Fig. 4*B*).

We also performed the same experiments with Smad2 mutants with altered membrane and protein-binding activity. For these experiments, we suppressed the expression of endogenous Smad2 in HeLa cells by siRNA (Fig. S2) and reintroduced mouse EGFP-Smad2 WT and mutants to the Smad2-suppressed cells, in order to circumvent the competition between the endogenous Smad2 and an exogenous Smad2 mutant with compromised activity. As shown in Figure 4*B*, W422A with reduced PI(4,5)P_2_ affinity showed little PM localization. In contrast, K420A with greatly reduced affinity for the TβRI peptide was able to migrate to the PM as fast as WT. These results indicate that the PI(4,5)P_2_-binding activity of Smad2 is essential for its TGF-β-stimulated PM recruitment, whereas its TβRI-binding activity is not important for the process. Also, the slow dissociation of K420A, which is similar to that of Smad2 WT after TβRI kinase inhibition, indicates that the weak interaction between K420A and TβRI severely suppresses Smad2 phosphorylation by TβRI.

Spontaneous PI(4,5)P_2_-dependent PM recruitment of Smad2 by TGF-β stimulation suggested that TGFβ stimulation might increase the PI(4,5)P_2_ concentration at the PM either globally or locally. We thus quantified PI(4,5)P_2_ in the PM by our ratiometric imaging analysis that allows spatiotemporally resolved PI(4,5)P_2_ quantification in live cells (36, 37, 38). As shown in Figure 4*C*, neither spatiotemporal distribution of PI(4,5)P_2_ nor the spatially averaged PI(4,5)P_2_ concentration was altered after TGF-β stimulation. We then explored the possibility that PI(4,5)P_2_ is locally enriched around the activated TGF-β signaling complex, which could not be visualized by our ratiometric imaging due to the limited spatial resolution of confocal imaging. For this purpose, we performed dual-color single molecule tracking analysis of TβRI and PI(4,5)P_2_, which allows for monitoring dynamic colocalization of the two molecules with higher spatiotemporal resolution than confocal imaging (21, 26, 39). Since endogenous PI(4,5)P_2_ cannot be directly tracked and commercially available fluorescent PI(4,5)P_2_ molecules are not suited for dual-color single-molecule tracking, we used the EGFP-phospholipase Cδ-pleckstrin homology domain (EGFP-PLCδ-PH) to track PI(4,5)P_2_ (36) (Fig. 4*D*). The same approach has been successfully used to demonstrate clustering of lipids, such as cholesterol and phosphatidylinositol-3,4,5-trisphsphate (PI(3,4,5)P_3_), near the PM-resident or -bound proteins (27, 38). Upon TGF-β stimulation, tetramethylrhodamine (TMR)-labeled SNAP-TβRI and EGFP-PLCδ-PH showed enhanced dynamic colocalization, as indicated by a longer dwell time of the TβRI-PLCδ-PH complex, which reached a maximum at 5 min after TGF-β stimulation (Fig. 4*D*). This indicates the local enrichment of PI(4,5)P_2_ in the vicinity of the activated TGF-β receptor complex. As expected, PI(4,5)P_2_ depletion eliminated colocalization of TMR-SNAP-TβRI and EGFP-PLCδ-PH before and after TGF-β stimulation (Fig. 4*D*). Most importantly, synchronization of the TβRI-PI(4,5)P_2_ colocalization (Fig. 4*D*) with the PM localization of Smad2 (Fig. 4*B*) supports the notion that local enrichment of PI(4,5)P_2_ around the activated TGF-β receptor complex triggers PM recruitment of Smad2.

We also performed dynamic colocalization analysis of TβRI and Smad2 (WT and mutants) to assess the importance of PI(4,5)P_2_ in functional interaction of Smad2 and TβRI in the activated TGF-β receptor complex (Fig. 4*E*). As described above, we suppressed the expression of endogenous Smad2 in HeLa cells by siRNA and reintroduced mouse EGFP-Smad2 WT and mutants to the Smad2-suppressed cells. TGF-β stimulation enhanced the colocalization time of TMR-SNAP-TβRI and EGFP-Smad2 WT, which peaked at 5 min after stimulation (Fig. 4*E*). Kinetics of their colocalization was similar to that of the PM localization of Smad2 (Fig. 4*B*), supporting the notion that binding of Smad2 to PI(4,5)P_2_ in the PM is immediately followed by its binding to TβRI. Under the same conditions, all mutants but E425K showed greatly reduced dwell times with TβRI after TGF-β stimulation (Fig. 4*E*). That is, W422A and Y426A with reduced PI(4,5)P_2_ affinity exhibit much reduced TGFβ-stimulated dynamic colocalization with TβRI. Similarly, R427A/R428A with reduced binding to both PI(4,5)P_2_ vesicles and the TβRI peptides (see Table 1) showed low response to TGFβ stimulation. Also, K420A with reduced binding to the TβRI peptides showed little response to TGFβ stimulation. In contrast, E425K with enhanced binding to both PI(4,5)P_2_ vesicles and the TβRI peptides had stronger interaction with TβRI in terms of both amplitude and duration (Fig. 4*E*). Collectively, these cellular biophysical measurements show that PI(4,5)P_2_-binding activity of Smad2 is essential for its PM recruitment and subsequent interaction with TβRI.

### The PI(4,5)P_2_-binding activity of Smad2 is essential for its nuclear transport and transcriptional activity

**Figure 5:**
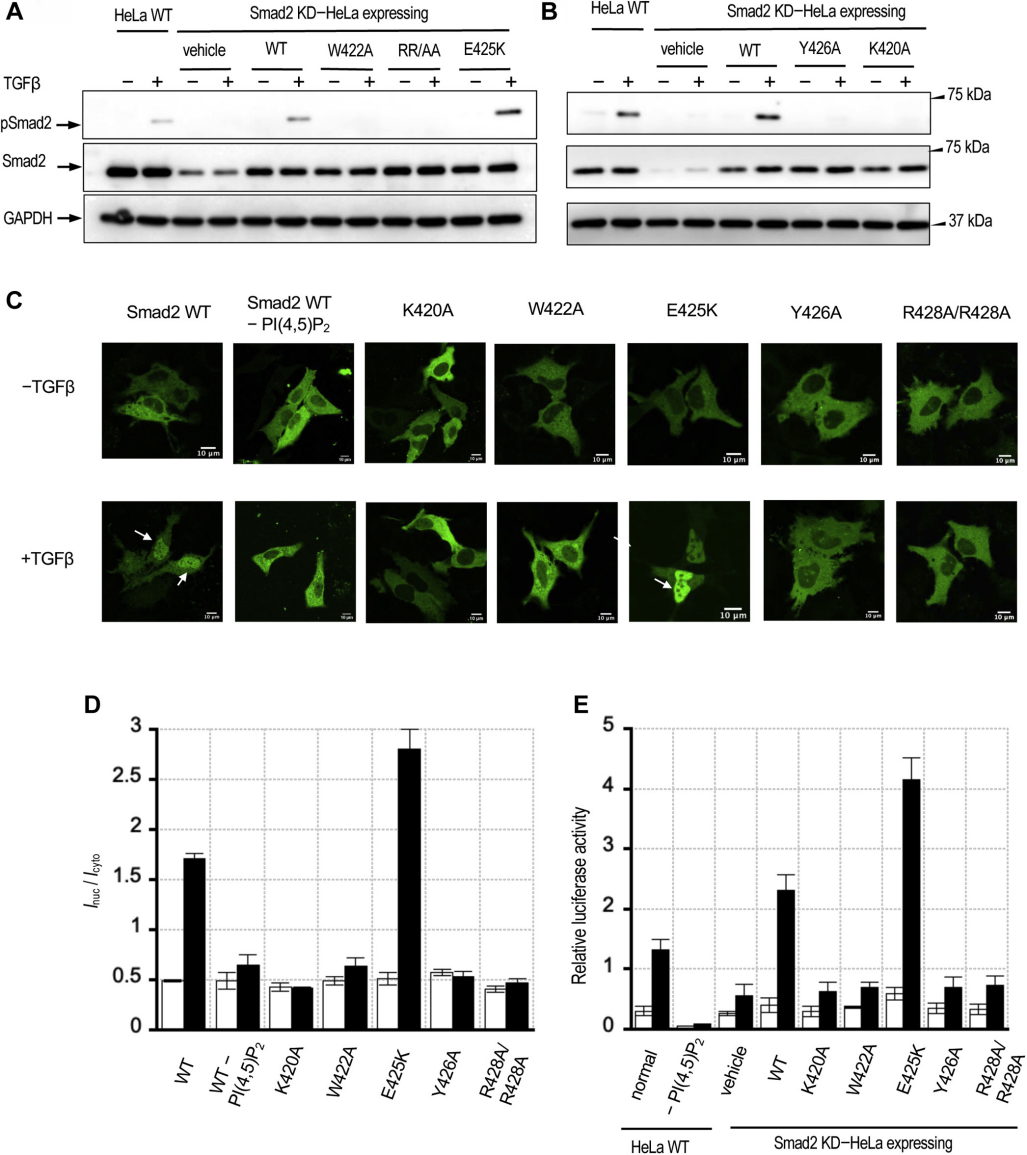
**Roles of PI(4,5)P**_**2**_**-dependent PM targeting of Smad2 in TGF-β signaling activities.***A* and *B*, phosphorylation of Smad2 (pSmad2) at S465 and S467 by TβRI was monitored before and 48 h after TGF-β stimulation (10 ng/ml) for HeLa WT cells and HeLa cells expressing mouse Smad2 WT, W422A, R427A/R428A (RR/AA), E425K, Y428A, and K420A (in this order), after suppression of endogenous Smad2 by siRNA (KD). Two separate gels were used to accommodate all mutants. An empty vector transfection (vehicle) was used for a negative control for each gel. Glyceraldehyde 3-phosphate dehydrogenase (GAPDH) was used as a gel loading control. Data in *A* and *B* are quantified in Fig. S3*A*. *C*, TGF-β stimulated (10 ng/ml for 1 h) nuclear translocation of mouse EGFP-WT, -K420A, -W422A, -E425K, -Y426A, and -R427A/R428A transfected into HeLa cells whose endogenous Smad2 was suppressed by siRNA. For Smad2-WT, the effect of PI(4,5)P_2_ depletion was measured. *Arrows* indicate nuclear accumulation of EGFP-WT and EGFP-E425K. Scale bars indicate 10 μm. *D*, quantification of (*C*). (*I*_nuc_/*I*_cyto_) was calculated from total nuclear and cytosolic fluorescence intensity values. *Blank* and *black bars* indicate values before and after TGF-β stimulation. Error bars indicate S.D. values from >3 measurements (*n* = 10). *E*, Smad2 transcriptional activity measured by the luciferase assay. *Blank* and *black bars* indicate values before and after TGF-β stimulation. Error bars indicate S.D. values from >3 measurements (*n* = 10).

To determine the physiological significance of the PI(4,5)P_2_-binding activity of Smad2, we systematically measured the effects of the PI(4,5)P_2_ depletion and Smad2 mutations on the phosphorylation, nuclear transport, and transcriptional activity of Smad2. As described above, we suppressed the expression of endogenous Smad2 in HeLa cells and reintroduced mouse EGFP-Smad2 WT and mutants. We first measured the phosphorylation of Smad2 WT and mutants in response to TGF-β stimulation (Fig. 5, *A* and *B*). TGF-β stimulation induces TβRI to phosphorylate S465 and S467 of Smad2 in the C-terminal end (1, 2). When EGFP-Smad2 WT was added back to Smad2-depleted HeLa cells, the degree of Smad2 phosphorylation was fully restored after TGF-β stimulation (Fig. 5, *A* and *B*). Under the same conditions, all PI(4,5)P_2_ binding-compromised mutants, W422A, Y426A, and R427A/R428A, were minimally phosphorylated where the lipid-binding gain-of-function mutant, E425K, was phosphorylated to a larger extent than WT (Figs. 5, *A* and *B* and S3*A*). Consistent with these results, PI(4,5)P_2_ depletion also greatly suppressed Smad2 phosphorylation (Fig. S3, *A* and *B*). K420A with compromised TβRI binding also showed little phosphorylation after TGF-β stimulation (Figs. 5*B* and S3*A*).

When we monitored the nucleocytoplasmic transport of EGFP-Smad2 proteins by confocal microscopy, we also found profound differences between Smad2 WT and mutants (Fig. 5, *C* and *D*). As reported previously (1, 2), a majority of EGFP-Smad2 WT molecules moved from the cytoplasm to the nucleus in response to TGF-β stimulation, as indicated by the shift in EGFP fluorescence intensity (Fig. 5, *C* and *D*). However, K420A, W422A, Y426A, and R427A/R428A did not show nuclear transport, whereas E425K was excessively transported to the nucleus (Fig. 5, *C* and *D*). Also, PI(4,5)P_2_ depletion abrogated the nuclear transport of Smad2 WT (Fig. 5, *C* and *D*).

We also measured the Smad2 transcriptional activity by the luciferase assay. Consistent with the results from the phosphorylation assay and nucleocytoplasmic transport imaging of Smad2, addition of Smad2 WT to Smad2-suppressed HeLa cells fully restored Smad2 transcriptional activity after TGF-β stimulation while exogeneous E425K showed higher transcriptional activity than exogeneous Smad2 WT (Fig. 5*E*). However, K420A, W422A, Y426A, and R427A/R428A showed much reduced activity even after TGF-β stimulation (Fig. 5*E*). Likewise, PI(4,5)P_2_ depletion abrogated TGF-β-induced Smad2 transcriptional activity in HeLa cells (Fig. 5*E*). To assess the role of SARA in Smad2 activation, we suppressed the expression of SARA in HeLa cells by siRNA and measured its effect on the Smad2 transcriptional activity (Fig. S4). Results show that SARA is not directly involved in Smad2 activation under our experimental conditions. Collectively, these results show that PI(4,5)P_2_-binding activity of Smad2 is important for the propagation of Smad-dependent TGF-β signaling pathways under physiological conditions.

## Discussion

Lipids play important regulatory roles in diverse receptor signaling pathways, including G-protein-coupled receptor (40) and growth factor signaling pathways (41), as many receptors and cytosolic signaling proteins that constitute signaling complexes directly interact with membrane lipids. While much is known about the mechanisms and physiological effects of TGFβ signaling (1, 2), little is known about the roles of lipids in TGFβ signaling. To our knowledge, only lipid implications reported so far are distinct compartmentalization of Smad-dependent and Smad-independent TGFβ signaling complexes in clathrin-coated pits (6) and caveolae (42) in the PM, respectively, and PI(3)P-dependent EE-localization of SARA as a Smad adaptor protein (7). Even in these cases, specific and crucial role of lipids in TGFβ signaling has not been demonstrated. The present study thus represents the first systematic and quantitative analysis of regulation of TGFβ signaling by membrane lipids. Our work establishes that Smad2 is a PI(4,5)P_2_-binding protein whose PM recruitment and activation during TGFβ signaling are specifically regulated by PI(4,5)P_2_. As such, Smad2 joins growing list of PI(4,5)P_2_-dependent cellular proteins that mediate diverse cellular processes, including cell signaling and membrane trafficking (16, 43, 44).

Smad2 binds PI(4,5)P_2_-containing membranes with affinity that is comparable to other reported PI(4,5)P_2_-binding proteins (45). It also has selectivity for PI(4,5)P_2_ over other PtdInsPs, which is conferred by the cationic pocket in the MH2 domain that can selectively recognize the PI(4,5)P_2_ headgroup. W422, Y426, R427, and R428 constitute the PI(4,5)P_2_-binding pocket, whereas K420 is located on the surface surrounding the rim of the pocket (Fig. 2*C*). Consistent with their molecular locations, mutations of W422, Y426, and R427, and R428 significantly and selectively reduce binding to PI(4,5)P_2_-containing membranes, whereas that of K420 modestly and nonselectively decreases binding of Smad2 to any anionic membranes. This systematic structural modeling and structure–function analysis clearly defines the membrane-binding surface and the PI(4,5)P_2_-binding pocket of Smad2.

Many cytosolic and PM-resident proteins have been shown to bind PI(4,5)P_2_ (43) but the spatially averaged concentration of available PI(4,5)P_2_ in the PM is low (*ca.* 1 mol%) compared with other phospholipids (36, 37, 38). Accordingly, a proportion of those cytosolic proteins that are prelocalized to the PM exclusively through PI(4,5)P_2_ binding is relatively low. It usually takes secondary interaction to recruit them to the PM to a large extent and for an extended period of time. The secondary interaction may be interaction with another lipid(s) (46) or a PM-resident protein(s) (17), which is typically triggered in a stimulus-dependent manner. Our dual-color single-molecule tracking analysis indicates that TGF-β stimulation induces local enrichment of PI(4,5)P_2_ around the activated TβRI (Fig. 4*D*). Although we do not fully understand the mechanism underlying this observation, which is currently under investigation, the fact that TGF-β-induced local clustering of PI(4,5)P_2_ near the activated TβRI (Fig. 4*D*) is synchronized with the TGF-β-stimulated PM localization of Smad2 (Fig. 4*B*) strongly supports the notion that PM recruitment of Smad2 is triggered and driven by the local enrichment of PI(4,5)P_2_.

**Figure 6:**
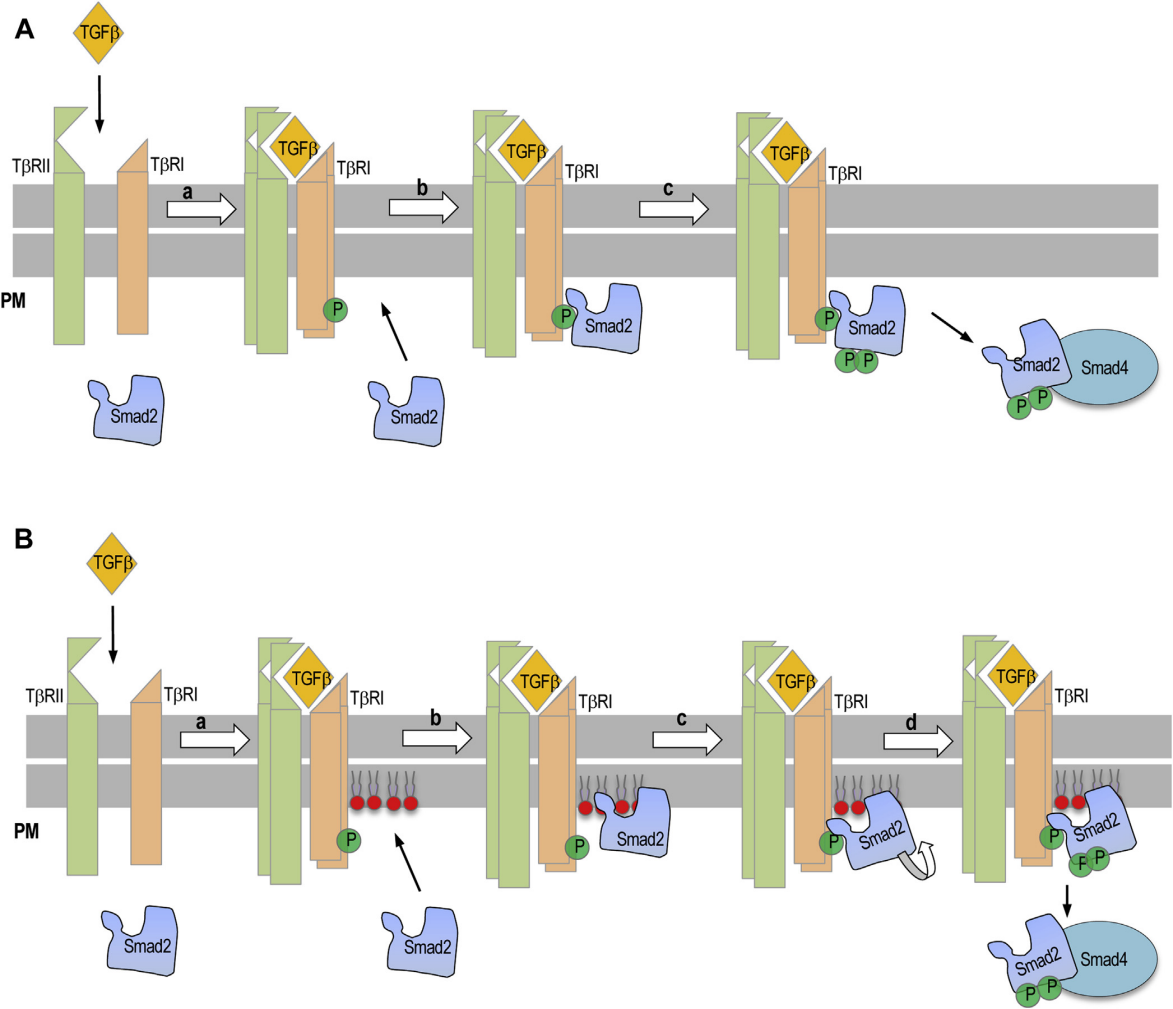
**The canonical and revisedmodels of PI(4,5)P**_**2**_**-dependent PM targeting and activation of Smad2.***A*, in the canonical model, a, TGF-β binding to TβRII induces heteromerization of TβRII-TβRI, phosphorylation of TβRI. b, Smad2 is then recruited to the PM by phosphorylated TβRI. c, TβRI-Smad2 binding leads to phosphorylation of S465 and S467, which promotes PM dissociation and Smad4 binding of Smad2 for nuclear translocation and transcriptional activity. *B*, in our proposed model, a, TGF-β binding to TβRII induces heteromerization of TβRII-TβRI, phosphorylation of TβRI, and local enrichment of PI(4,5)P_2_. b, Smad2 is recruited to the PM by locally enriched PI(4,5)P_2_. c, PM-anchoring of Smad2 by PI(4,5)P_2_ facilitates its interaction with phosphorylated TβRI with potential reorientation of the PM-bound Smad2 molecule. d, TβRI-Smad2 binding leads to phosphorylation of S465 and S467, which promotes PM dissociation and Smad4 binding of Smad2 for nuclear translocation and transcriptional activity.

Our study also reevaluates the importance of the Smad2-TβRI binding in the TGF-β-triggered PM recruitment of Smad2. It has been generally thought that Smad2 is recruited to the activated TGF-β receptor complex through the Smad2-TβRI interaction (1, 2) (see Fig. 6*A*). Due to lack of high-resolution structures of the Smad2 (or Smad3)-TβRI complex, the Smad2-TβRI-binding interface has been mostly deduced from mutational studies (3, 28, 29). In particular, the L3 loop of Smad2 MH2 domain, where its PI(4,5)P_2_-binding site is located, was assigned to interact with the L45 loop of TβRI, whereas the H1 helix of Smad2 with the phosphorylated GS domain of TβRI. However, these earlier studies were not based on systematic structure–function analysis of purified proteins by direct and quantitative binding measurements, leaving the possibility of misassignment. Our systematic structure–function study of purified Smad2 WT and mutants by direct and quantitative binding analysis suggests that the L3 loop residues in the MH2 domain of Smad2 interact more closely with the GS domain than with the L45 loop (see Fig. 3). Although TβRI-derived peptides may not fully represent the PM-resident TβRI, the striking positional specificity observed in our study, *i.e.*, much lower affinity of K420A (*i.e.*, tenfold lower affinity than the WT) for the GS peptide than the doubly mutated R427A/R428A (*i.e.*, twofold lower affinity than the WT) (see Fig. 3*A*), supports the specific nature of our Smad2-GS peptide binding. Also, the micromolar affinity of Smad2-GS peptide binding (see Table 1) is comparable to that for SH2 domain-phosphotyrosine peptide binding (47, 48). Most importantly, little to no effect of the K420A mutation, which reduces the affinity of Smad2 for the GS peptide by an order of magnitude, on the TGF-β-stimulated PM targeting of Smad2 points to the fact that the Smad2-TβRI binding does not significantly contribute to the PM localization process of Smad2. Our peptide-binding study in the presence of PI(4,5)P_2_-containing vesicles shows that the partial overlap of the membrane- and TβRI-binding sites in the Smad2 MH domain interferes with coincident binding of Smad2 to membrane lipids and TβRI. Based on these results, we propose that Smad2 is initially recruited to the PM by PI(4,5)P_2_ binding (Fig. 6*B*). PI(4,5)P_2_-mediated PM anchoring of Smad2 should then greatly facilitate its interaction with activated TβRI at the PM due to reduction in dimensionality (14, 49). Synchronization of the TβRI-PI(4,5)P_2_ colocalization (Fig. 4*D*), the PM translocation of Smad2 (Fig. 4*B*), and the dynamic Smad2-TβRI colocalization (Fig. 4*E*) indicate that Smad2-TβRI binding immediately follows the PI(4,5)P_2_-mediated PM recruitment of Smad2. The Smad2-TβRI binding will then lead to Smad2 phosphorylation by TβRI, which in turn leads to dissociation of Smad2 from the TGF-β receptor complex and ensuing TGF-β signaling processes. The importance of TβRI-mediated phosphorylation of Smad2 in PM dissociation of Smad2 is supported by slow membrane dissociation of Smad2 caused by kinase inhibition of TβRI and K420A mutation of Smad2 that suppresses the Smad2-TβRI interaction, respectively (Fig. 4*B*).

Our immunoblotting analysis, confocal imaging of nucleocytoplasmic dynamics, and TGF-β transcriptional activity measurements of Smad2 WT and mutants (Fig. 5) show that PI(4,5)P_2_-dependent PM recruitment of Smad2 leads to functional activation of Smad2. In particular, an excellent correlation between the relative PI(4,5)P_2_ affinity of mutants and their phosphorylation by TβRI, nuclear translocation, and transcriptional activity demonstrates that PI(4,5)P_2_-dependent PM recruitment of Smad2 is a critical step in the TGFβ signaling activity of Smad2.

Our work also provides new insight into the subcellular spatiotemporal dynamics of the TGF-β receptor signaling complex. It has been debated whether the main site of Smad-mediated TGF-β signaling is the PM or EEs (6, 7, 8, 9, 10, 11, 12, 13). It should be noted that the notion that the activated TGF-β receptor signaling complex is primarily located at the EEs is not based on direct visualization of the complex at EEs. Rather, it is indirectly supported by the potential localization of the complex in endocytic clathrin-coated pits and the potential involvement of PI(3)P-binding SARA in membrane recruitment and activation of Smad2/3 (6, 7). However, both of these reports have been experimentally challenged (8, 9, 10, 11, 12, 13). Also, our results show that SARA is not directly involved in Smad2 activation under our experimental conditions. Our SPR studies show that although Smad2 prefers PI(4,5)P_2_ to PI(3)P, it can still interact favorably with PI(3)P (see Fig. 1*B* and Table 1). Thus, if the activated TGF-β signaling complex is endocytosed and moves to EEs, Smad2 should continue to be recruited to the signaling complex at EEs by targeting PI(3)P in lieu of PI(4,5)P_2_ even in the absence of SARA. Further studies are necessary to fully understand the mechanism and physiological significance of the Smad-dependent TGF-β signaling process at EEs.

All Smad proteins have structurally similar MH2 domains, and the key PI(4,5)P_2_-binding residues, W422, Y426, and R428 of Smad2, are fully conserved among Smad proteins (2) (Fig. S5). Thus, most, if not all, of the Smad proteins are expected to bind membrane lipids, with some degree of selectivity for PI(4,5)P_2_ or another lipid, depending on the topology of their lipid-binding site. Further studies on Smad–lipid interactions will shed light on the roles of PI(4,5)P_2_ and other lipids in signaling activities of various Smad proteins. Smad3 has a structurally similar MH2 domain to Smad2 and consequently binds PI(4,5)P_2_-containing membranes almost as well as Smad2 (see Table 1). These structural and functional properties of Smad3 lead us to propose that the spatiotemporal dynamics and functional activity of Smad3 also depend on its PI(4,5)P_2_-binding activity. Although functional differences have been reported between Smad2 and Smad3 (2, 23, 30, 50), it is not fully understood as to whether they compensate, cooperate, or antagonize with each other during Smad-dependent TGFβ signaling. Therefore, it is premature to speculate on how common PI(4,5)P_2_-binding activity of Smad2 and Smad3 would influence the overall Smad-dependent TGFβ signaling pathway. Again. further studies are needed to fully address this important question.

## Experimental procedures

### Materials

POPC and POPS were purchased from Avanti Polar Lipids.1,2-dipalmitoyl derivatives of PI(4,5)P_2_, PI(3,4)P_2_, phosphatidylinositol-3,4,5-bisphosphate (PI(3,4,5)P_3_), and PI(3)P were from Cayman Chemical Co (Cat no. 10008115). TGF-β1 was purchased from Millipore sigma (Cat no. T7039-2UG; lot no. SLBZ7941). Custom-designed peptide was purchase from AlanScientific. siRNAs for human Smad2 were purchased from Integrated DNA Technologies and the transfection reagent JetPRIME was from Polyplus transfection. Reporter plasmids and the dual luciferase reporter assay system (Cat no. E1960) were from Promega. Antibodies against Smad2 (Cat no. 5339S), phospho-Smad2 (pS465/pS467) (Cat no. 18338S), and glyceraldehyde 3-phosphate dehydrogenase (GAPDH) (Cat no. 5174S) were purchased from Cell Signaling Technologies. The anti-SARA antibody was from Santa Cruz Biotechnology. TβRI kinase inhibitor SB-431542 (Cat no. HY-10431) was from MedChemExpress.

### Bacterial expression and purification of Smad proteins

All Smad2 proteins were produced using the pET-30a vector with an N-terminal His_6_-tag. The construct was transformed to *E. coli* BL21 RIL codon plus cells (Stratagene) for the bacterial expression. A preculture solution was prepared from a single colony in 10 ml of LB media with 50 μg/ml kanamycin and incubated in a shaker at 37 °C overnight or until it got cloudy. The 5 ml portion of the preculture was transferred to 500 ml of the main culture medium with μg/ml kanamycin, and the mixture was incubated in a shaker at 37 °C until the absorbance at 600 nm reached 0.6. Protein expression was then induced at 19 °C with 0.5 mM isopropyl β-d-1-thiogalactopyranoside for 16 h. The culture medium was pelleted by centrifugation at 4000*g* for 10 min. Cell pellets were stored at −80 °C until use. The cells were resuspended with 20 ml of the lysis buffer (50 mM Tris-HCl, pH 7.9, with 300 mM NaCl, 10 mM Imidazole, 10% glycerol, 1 mM phenylmethanesulfonylfluoride, and 1 mM dithiothreitol) and lysed by sonication. The lysate was centrifuged at 44,000*g* for 30 min and the clear supernatant was mixed with 1 ml of Ni-NTA agarose resin (Marvelgent Biosciences Inc), and the mixture was incubated 4 °C for 2 h with gentle mixing. The resin was washed with consecutively 100 ml of the wash buffer 1 (50 mM Tris HCl, pH 7.9, with 300 mM NaCl, 20 mM imidazole), 50 ml of the wash buffer 2 (50 mM Tris HCl, pH 7.9, with 500 mM NaCl, 20 mM imidazole), and 100 ml of the wash buffer 3 (20 mM Tris HCl, pH 7.9 with 160 mM NaCl, 40 mM imidazole). The protein was then eluted from the resin with 1 ml of the elution buffer (50 mM Tris HCl, pH 7.9, with 300 mM NaCl, 300 mM imidazole). The protein concentration was determined by the Bradford assay.

### Surface plasmon resonance (SPR) analysis

All SPR measurements were performed at 23 °C in 20 mM Tris, pH 7.4, containing 0.16 M NaCl using a lipid-coated L1 chip in the BIACORE X-100 system (GE Healthcare) as described previously (19, 38). LUVs of POPC/POPS/PtdInsP (77:20:3) and POPC were used as the active surface and the control surface, respectively. Sensorgrams were collected for both membrane association and dissociation but the only the association phases were further analyzed because the dissociation phases were often too slow for analysis. For PtdInsP selectivity determination, sensorgrams were normalized by converting RU values into RU/RU_max_ values that represent the fraction of the membrane-bound protein molecules at a given protein concentration where RU_max_ indicates the maximal RU value when a given lipid surface is saturated with the protein molecules. RU_max_ for each PtdInsP-containing LUV was estimated by employing 1 μM of Smad2. For Kd determination, normalized sensorgrams obtained at varying protein concentrations were analyzed assuming a Langmuir-type binding between the protein (P) and protein-binding sites (M) on vesicles (that is, P + M↔PM). The RU/RU_max_ values were plotted against the protein concentrations (Po), and the Kd was established by nonlinear least squares analysis of the binding isotherm using the equation, RU/RU_max_ = 1/(1 + Kd/Po). The flow rate was maintained at 30 μl/min for both association and dissociation phases.

### *In silico* molecular docking analysis

The potential binding modes of the PI(4,5)P_2_ and Smad2 protein were predicted using Autodock4 software (The Scripps Research Institute) (51). An 1,2-dibutyroyl derivative of PI(4,5)P_2_ was drawn and energy-minimized with Chem Draw ultra and Chem 3D ultra, respectively. The structure was saved as sdf file and the protein data bank file was generated using OpenBabel2.3.1 software (52). The crystal structure of Smad2 (protein data bank ID: 1DEV) (24) was used for docking analyses. The macromolecule was then prepared by removing the chain D and water molecules and adding hydrogens and Kollman charges, and saved in PDBQT format for the Autodock4 program. A cube-shaped grid coordinates (dimension: *x* = 78, *y* = 78, *z* = 78 and center-*x* = 5.917, *y* = −14.972, *z* = −10.75) were set to cover the binding site of Smad2. To run the docking, the parameters were kept as default. Finally, the generated dlg file was analyzed and the lowest energy-binding conformation was considered as the best docking pose. The docking pose was exported and illustrated using PyMOL (The PyMOL Molecular Graphics System, Version 2.0 Schrödinger, LLC).

### Membrane translocation analysis of Smad2

For calculating the relative ratio between the fluorescence intensity of PM (*F*_m_) and the cytosolic area (*F*_c_), the line profile function in Image-Pro Plus software (Media Cybernetics) was used. The detailed calculation method was described as reported (53). Briefly, a line was drawn across the cell image, and the distance-dependent intensity plot with two peaks indicating PM was obtained. If the nucleus region has a strong signal, the line was drawn over the nonnuclear region. *F*_m_ was calculated by averaging the two PM peaks, whereas *F*_c_ was calculated by averaging the intensity of the area between the two peaks.

### Single-molecule tracking

Single-molecule imaging was performed using a custom-built total internal reflection fluorescence microscope as described previously. HeLa cells were plated on the 8-well chambered coverglass (Lab-Tek, Thermo Fisher Scientific) at the density of 1 × 10^5^ for 24 h, EGFP-Smad2 or mutants and SNAP-tagged TβRI were cotransfected into cells using the jetPRIME system (Polyplus-transfection) according to the manufacturer's protocols. The transfected HeLa cells were serum starved for 16 h and subsequently labeled with SNAP-Cell tetramethylrhodamine (TMR)-Star (New England Biolabs). Labeled cells were washed to remove the free dye, stimulated with 10 ng/ml TGF-β, the two protein molecules were simultaneously tracked and analyzed as described. The images were spatially corrected following the algorithm described previously (39). All particle tracking, data analysis, and image processing were carried out with in-house programs written in MATLAB. Colocalization analysis of two molecules was performed with a fixed threshold criterion (*i.e.*, <400 nm) for colocalization (39). The same size of PM surface was analyzed for each data. The percentage of Smad2 molecules spending a given colocalization time (>0.2 s) with TGF-β RI on the PM of HeLa cells was calculated from the total number of Smad2 molecules and displayed as a histogram. Data were fit into a single exponential decay equation (*i.e.*, *P* = *P*_o_ *e*^*−kt*^) to determine the dissociation rate constant (*k*) values by nonlinear least-squares analysis and the half-life values of colocalization were calculated as *ln*2/*k*. In total, 10 to 20 images were analyzed for each data point.

### Peptide-binding measurements

The fluorescein-6-aminohexanoyl (F-Ahx)-labeled TβRI GS region peptide (F-Ahx-YDMTTpSGpSGpSGLPLL) was dissolved in dimethyl sulfoxide to yield 1 mg/ml stock solution, F-Ahx-labeled TβRI L45 loop region peptide (F-Ahx-ADNKDNGT) was dissolved in a 3:1 mixture of water and acetonitrile to yield 2.5 mg/ml stock solution. Both peptide solutions were diluted to 1 to 10 μM with 20 mM Tris buffer, pH 7.9, containing 160 mM NaCl for binding studies. In total, 300 μl of the Smad2 (WT or mutant) solution (0–250 μM) was added to a series of 1.5 ml microcentrifuge containing the peptide solution (2.5 μM). After 10-min incubation in the dark, the mixture was transferred to a quartz cuvette with 2-mm path length and fluorescence anisotropy (*r*) was measured with excitation and emission wavelengths set at 485 and 535 nm, respectively, using Horiba Flurolog-3 spectrofluorometer. Since *P*_*o*_ >> *Pep*_*o*_ under our conditions, the *K*_*d*_ for the Smad2-peptide binding was determined by the nonlinear least-squares analysis of the binding isotherm using the Equation 1:

(1) $$Pep_{bound}/Pep_{0}=\Delta r/\Delta r_{max}=\frac{1}{1+k_{d}/P_{0}}$$

where *Pep*_*bound*_, *Pep*_*0*_, and *P*_*0*_ indicate the concentration of bound peptide, total peptide, and total Smad2, respectively, and *Δr* and *Δr*_*max*_ are the anisotropy change for each *P*_*0*_ and the maximal *Δr*, respectively.

### siRNA knockdown

HeLa cells were plated on 6-well or 48-well plates at the density of ∼1.5 × 10^5^ or 1.25 × 10^4^ for 24 h and 30 nM human Smad2 (or SARA) DsiRNAs (IDT: see sequences below) were transfected into cells using the jetPRIME system (Polyplus transfection) according to the manufacturer's protocols. After transfection with siRNA for 24 h, the medium was exchanged with a fresh one and cells were transfected again with 1 μg/ml of the mouse Smad2 expression vector. In total, 24 to 48 h after transfection, cells were used for further treatment or Western blot analysis. The sequences of DsiRNAs are as follows: SMAD2-siRNA1 (sense: 5′-GGCAUUGAUACUUAGACAUAUCAAA-3′; antisense: 5′-UUUGAUAUGUCUAAGUAUCAAUGCCUU-3′); SMAD2-siRNA2 (sense: 5′-CUGCUUAGGUUUACUCUCCAAUGTT-3′; antisense: 5′-AACAUUGGAGAGUAAACCUAAGCAGAA-3′); SMAD2-siRNA3 (sense: 5′-CUGCUUAGGUUUACUCUCCAAUGTT-3′; antisense: 5′-AACAUUGGAGAGUAAACCUAAGCAGAA-3′); SARA-siRNA1 (sense: 5′-ACAGUUUCUUCUACUUUAUUGGATA-3′; antisense: 5′-UAUCCAAUAAAGUAGAAGAAACUGUUU-3′); SARA-siRNA2 (sense: 5′-AAGCAACCUUCUAAUCUUAAACUTC-3′; antisense: 5′-GAAGUUUAAGAUUAGAAGGUUGCUUGG-3′).

### Western blot analysis

Transfected cells or treated cells were lysed in the cell lysis buffer (20 mM Tris-HCl, pH 7.5, containing 150 mM NaCl, 1 mM Na_2_EDTA, 1 mM EGTA, 1% Triton-X, protease inhibitors, and phosphatase inhibitors) (1 mM Na_3_VO_4_, 1 mM NaF, 1 μg/ml leupeptin, 1 mM phenylmethanesulfonylfluoride, 1.5 mM benzamidine, and 2 μg/ml pepstatin). The total protein concentration of the cell lysate was determined by the Pierce BCA protein assay kit (Thermo scientific). The same amounts of proteins were loaded onto a polyacrylamide gel to run sodium dodecyl sulfate–polyacrylamide gel electrophoresis. Proteins were separated and transferred to a polyvinylidene difluoride membrane. The membrane was blocked with 5% bovine serum albumin for 1 h and incubated overnight at 4 °C with various antibodies (1:1000 dilution for all antibodies). After the unbound antibodies were removed by washing with 0.1% Tris buffer saline with 0.1% Tween20, the membranes were incubated with the horseradish peroxidase secondary antibody (1:5000 dilution) for 1 h at room temperature. The membranes were washed three more times with 0.1% Tris buffer saline with 0.1% Tween20 to remove the unbound horseradish peroxidase secondary antibody before imaging. The chemiluminescence intensity of protein bands in the gel was analyzed and documented by the Azure 500Q Imaging System.

### PI(4,5)P_2_ depletion from the plasma membrane

The plasmid for dual expression of Lyn-iRFP-FKBP12 and FRB-Inp54 was prepared by subcloning the genes encoding pPBH-TRE_tight_-Lyn-iRFP-FKBP12 and pCMV-FRB-Inp54 into a PiggyBac vector using In-Fusion Cloning Kit. The pCMV-FRB-Inp54 expression vector was controlled by the Tet-On system for reduced basal expression (54). The resulting dual expression plasmid (1.5 μg) and the recombination helper plasmid pSPB-Transposase (0.6 μg) were transfected into 70 to 80% confluent HeLa cells plated in a 6-well plate using the JetPRIME system (Polyplus-transfection) according to the manufacturer’s protocol. Cells in a separate well were kept without transfection as a control. After 24 h transfection, the growth media were replaced with the selection media (Dulbecco's modified Eagle's medium with 10% fetal bovine serum, 200 mg/ml hygromycin, 1% penicillin and streptomycin). The growth medium was replaced every other day until the cells in the control well were completely dead. Successful transfection and stable expression of Lyn-iRFP-FKBP12 and FRB-Inp54 were confirmed with the iRFP signal on the cell membrane by confocal microscopy. These stably transfected cells were maintained in the growth media containing 100 mg/ml hygromycin. PI(4,5)P_2_ depletion in these cells was induced by 1 μM of rapamycin and confirmed by ratiometric PI(4,5)P_2_ imaging.

### Ratiometric PI(4,5)P_2_ imaging analysis

The ratiometric PI(4,5)P_2_ sensor (DAN-eENTH) was prepared and calibrated using PM-mimetic giant unilamellar vesicles as described previously (36, 37, 38). DAN-eENTH was microinjected into HeLa cells, and the PI(4,5)P_2_ concentration in the PM was determined as described (36, 37, 38). The three-dimensional display of local lipid concentration profile was calculated using the Surf function in MATLAB.

### Quantitative TIRF microscope imaging analysis

Quantitative TIRF microscopy imaging of PM localization of EGFP tagged Smad2 WT and mutants was performed using custom-built internal reflection fluorescence microscope. WT HeLa cells or PI(4,5)P_2_ depleted HeLa cells were plated on the 8-well chambered cover glass (Lab-Tek, Thermo Fisher Scientific) at the density of 1 × 10^5^ for 24 h. Endogenous Smad2 was suppressed using SiRNA and EGFP-Smad2 or mutants were transfected into cells using the jetPRIME system (Polyplus-transfection) according to the manufacturer's protocols. The transfected HeLa cells were serum starved for 16 h prior imaging. PM-bound EGFP-Smad2 fraction was monitored before and after stimulation with 10 ng/ml TGF-β up to 15 min. For EGFP-Smad2 WT, the effect of TβRI inhibition was monitored by incubating cells with 10 μM of SB-431542 for 36 h. Image analysis was performed using Image J software. Mean intensity of the EGFP-Smad2 signal was calculated for each time point, and final graph was plot by subtracting the mean intensity at 0 min from the mean intensity of each time point. More than ten cells were analyzed for each data point.

### Nuclear translocation analysis of SMAD2

The ratio of nuclear to cytoplasmic protein was calculated from the fluorescence intensity of nucleus (*I*_nuc_) and the cytoplasm (*F*_cyto_) in cross-section images of cells using the line profile function of Image-Pro Plus software (Media Cybernetics). Briefly, at least five different lines were drawn across the cross-sectional image of each cell and the average *I*_nuc_ and *I*_Cyto_ values were calculated along the lines. Typically, more than ten cell images were analyzed for each data set to determine the average and SD values.

### Single-molecule imaging analysis

Single-molecule imaging was performed using a custom-built total internal reflection fluorescence (TIRF) microscope as described previously. HeLa cells were plated on the 8-well chambered coverglass (Lab-Tek, Thermo Fisher Scientific) at the density of 1 × 10^5^ for 24 h, and EGFP-Smad2 and SNAP-tagged TβRI were cotransfected into cells using the jetPRIME system (Polyplus-transfection) according to the manufacturer's protocols. The transfected HeLa cells were serum starved for 16 h and subsequently labeled with SNAP-Cell tetramethylrhodamine (TMR)-Star (New England Biolabs). Labeled cells were washed to remove the free dye, stimulated with 50 ng/ml TGF-β, the two protein molecules were simultaneously tracked and analyzed as described. The images were spatially corrected as described previously (39). All single-molecule tracking, data analysis, and image processing were carried out with in-house programs written in MATLAB. Colocalization analysis of two molecules was performed with a fixed threshold criterion (*i.e.*, <400 nm) for colocalization (39). The same size of PM surface was analyzed for each data. The percentage of Smad2 molecules spending a given colocalization time (>0.2 s) with TβRI on the PM of HeLa cells was calculated from the total number of Smad2 molecules and displayed as a histogram. Data were fit into a single exponential decay equation (*i.e.*, *P* = *P*_o_ *e*^*−kt*^) to determine the dissociation rate constant (*k*) values by nonlinear least-squares analysis and the half-life values of colocalization were calculated as *ln*2/*k*. In total, 50 to 100 images were analyzed for each data point.

### Dual luciferase reporter assay for Smad2 activity

HeLa cells were plated on 6-well plates at the density of ∼1.5 × 10^5^ and treated with siRNA as described above. The reporter gene containing three TGF-β/activin response element (ARE) coupled with the luciferase gene (pGL2 3ARE-Lux: Addgene) was used for enhanced activity (55). On translocation into the nucleus, receptor-activated Smad2 associates with the DNA-binding protein FAST1 to form a transcriptional complex on the ARE of the *Mix.2* promoter. After 24 h, the cell media were replaced with fresh media and cells were cotransfected with 375 ng pGL2 3ARE-Lux experimental vector, 37.5 ng pGL4.73[hRluc/SV40] internal control vector (Promega), 337.5 ng FAST1, and 750 ng of pcDNA 3.1 plasmid harboring Smad2 WT or mutant using the jetPRIME transfection system (Polyplus transfection) according to the manufacturer's protocols. After 24 h of transfection, cells were placed in fresh media and stimulated with 10 ng/ml TGF-β for 48 h. Then the cells were washed with phosphate buffer saline, and the dual luciferase reporter assay was performed according to the manufacturer's protocols (Promega). All 3ARE-Luc activity values were normalized using the pGL4.73[hRluc/SV40] values as a reference.

### Quantification and statistical analysis

All imaging data analysis and image processing were carried out with in-house programs written in MATLAB. The number of experiments, the number of total cells analyzed (*n*), and significance are reported in the figure legends. Sample sizes for cellular imaging and assays were chosen as the minimum number of independent observations required for statistically significant results.

## Data availability

All described data are contained within this manuscript.

## Supporting information

This article contains supporting information.

## Conflict of interest

The authors declare that have no conflicts of interests with the contents of this article.
