# Supplementary material for: Phosphoinositide-binding activity of Smad2 is essential for its function in TGF-β signaling
Source: J Biol Chem. 2021 Oct 14;297(5):101303. doi: 10.1016/j.jbc.2021.101303 (PMC8567202; doi:10.1016/j.jbc.2021.101303)
Supplement: Supplemental Figures S1–S5 [file mmc1.pdf]

SUPPLEMENTAL INFORMATION

Title: Phosphoinositide binding activity of Smad2 is essential for its function in TGF-β signaling

Authors: Pawanthi Buwaneka, Arthur Ralko, Sukhamoy Gorai, Ha Pham, and Wonhwa Cho*

Departments of Chemistry, University of Illinois at Chicago, Chicago, IL 60607, U.S.A.

**Corresponding author: Wonhwa Cho, Department of Chemistry, University of Illinois at Chicago, Chicago, IL 60607, USA;* E-mail: wcho@uic.edu

Supplemental Fig. S1. Confocal images of endogenous Smad2 and exogenously expressed EGFP-Smad2 in HeLa cells before (A) and 10 min after (B) TGF-β stimulation.
Endogenous Smad2 images were obtained by immunostaining using the Smad2-specific antibody. 10 ng/ml TGF-β was used for stimulation. Scale bars indicate 10 μm.

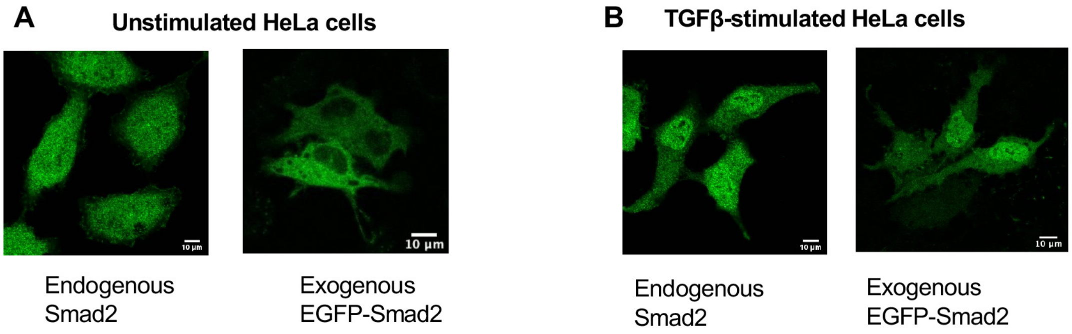

Supplemental Fig. S2. Suppression of endogenous Smad2 expression in HeLa cells by siRNA.
Western blot analysis showing that expression of endogenous Smad2 in HeLa cells was successfully suppressed by siRNA. 30 nM siRNA-3 was routinely used for knockdown experiments. For re-introduction of mouse Smad2 to these cells, see Fig. 5A. Glyceraldehyde 3-phosphate dehydrogenase (GAPDH) was used as a gel loading control.

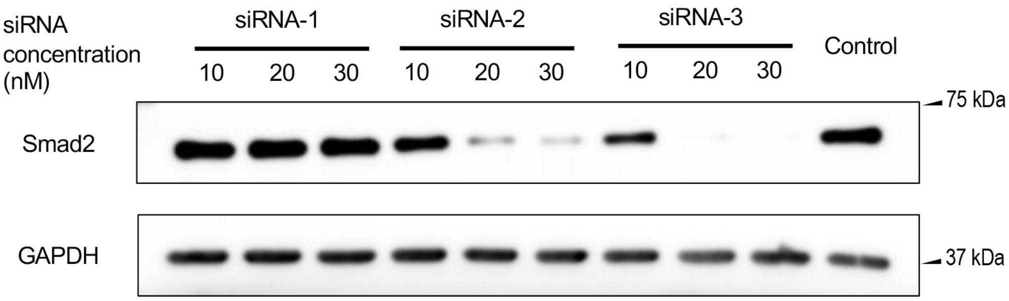

Supplemental Fig. S3. Roles of PI(4,5)P_2 binding of Smad2 in Smad2 phosphorylation.
(A) Quantification of Fig. 5A-B. The ratio of the intensity of a pSmad2 band to that of a total Smad2 band was calculated for each lane from the gels. Blank and black bars indicate values before (−) and after (+) TGF-β stimulation. Error bars indicate S.D. values from 3 measurements. (B) Phosphorylation of Smad2 (pSmad2) by TβRI was monitored before and 1 h after TGF-β stimulation (10 ng/ml) for HeLa WT cells before (+) and after (−) PI(4,5)P_2 depletion. GAPDH was used as a gel loading control.

A

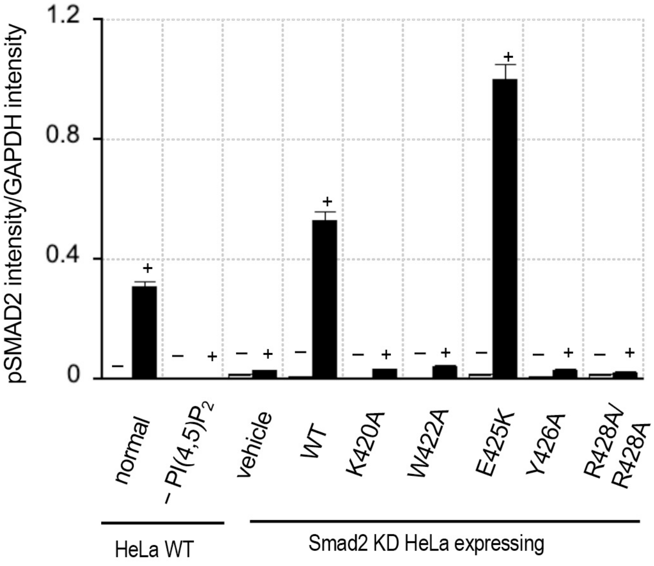

B

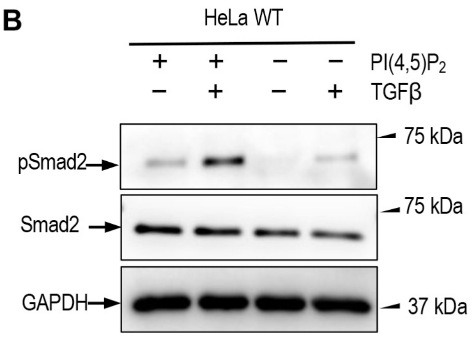

Supplemental Fig. S4. Effect of suppression of endogenous SARA expression in HeLa cells on the Smad2 transcriptional activity.

(A) Effects of suppression of endogenous SARA expression in HeLa cells by two siRNA's on the Smad2 transcriptional activity. 30 nM each of siRNA1 and 2 was used for knockdown experiments. (B) Western blot analysis showing that expression of endogenous SARA in HeLa cells was successfully suppressed by siRNA's. GAPDH was used as a gel loading control.

A

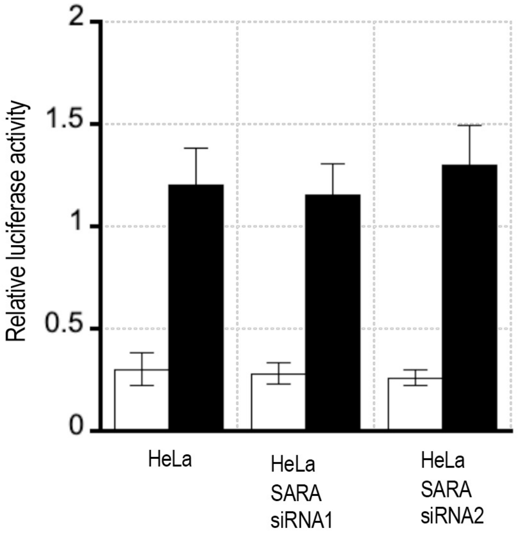

B

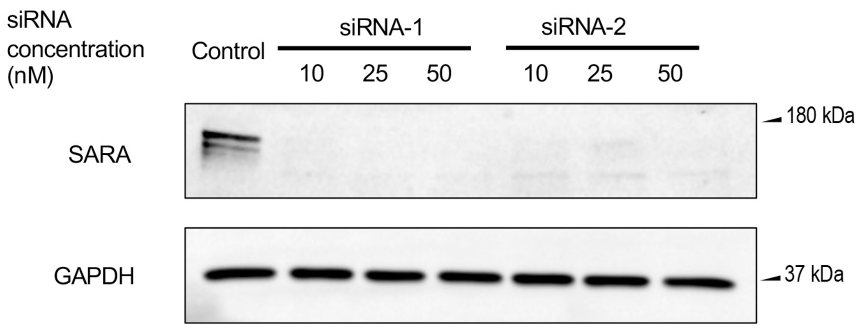

Supplemental Fig. S5. Multiple sequence alignment of mouse Smad MH2 domains

The analysis was performed by CLUSTAL Omega. Essential PI(4,5)P_2-binding residues of Smad2 (highlighted in red) are absolutely conserved among Smads. The asterisk (*), colon (:), and dot (.) indicate identical amino acid residues, conserved substitution, and semi-conserved substitutions, respectively.

```
SMAD1      --M-MAPP-LPAEISRGDVQAVA--YEEPKHWCSIVYYELNNRVGEAFHA--SSTSVLVD     297
SMAD2      SPAELSPTTLSPVNHSLDLQPVT--YSEPAFWCSIAYYELNQRVGETFHA--SQPSLTVD     300
SMAD3      SP-NLSPNPMSPAHNNLDLQPVT--YCEPAFWCSISYYELNQRVGETFHA--SQPSMTVD     226
SMAD4      PPMPPHPGHYWPVHNELAFQPPISNHPAPEYWCSIAYFEMDVQVGETFKVPSSCPVVTVD     350
SMAD5      --SNMIPQ-TMPSISSRDVQPVA--YEEPKHWCSIVYYELNNRVGEAFHA--SSTSVLVD     297
SMAD6      ---ITAPGEFSD-------ASMSPDATKPSHWCSVAYWEHRTRVGRLYAVYDQA----VS     356
SMAD7      ---YLAPGGLSD-------SQLLLEPGDRSHWCVVAYWEEKTRVGRLYCVQEPS----LD     284
SMAD8      ---------SDSPYQHSDFRPVC--YEEPQHWCSVAYYELNNRVGETFQA--SSRSVLID     260
                                         .** : *:*   :**. : .        :.

SMAD1      GFTDPSNNKNRFCLGLLSNVNRNSTIENTRRHIGKGVHLYYVG-GEVYAECLSDSSIFVQ     356
SMAD2      GFTDPSN-SERFCLGLLSNVNRNATVEMTRRHIGRGVRLYYIG-GEVFAECLSDSAIFVQ     358
SMAD3      GFTDPSN-SERFCLGLLSNVNRNAAVELTRRHIGRGVRLYYIG-GEVFAECLSDSAIFVQ     284
SMAD4      GYVDPSG-GDRFCLGQLSNVHRTEAIERARLHIGKGVQLECKGEGDVWVRCLSDHAVFVQ     409
SMAD5      GFTDPSNNKSRFCLGLLSNVNRNSTIENTRRHIGKGVHLYYVG-GEVYAECLSDSSIFVQ     356
SMAD6      IFYDLPQ-GSGFCLGQLNLEQRSESVRRTRSKIGFGILLSKEP-DGVWAYNRGEHPIFVN     414
SMAD7      IFYDLPQ-GNGFCLGQLNSDNKSQLVQKVRSKIGCGIQLTREV-DGVWVYNRSSYPIFIK     342
SMAD8      GFTDPSNNRNRFCLGLLSNVNRNSTIENTRRHIGKGVHLYYVG-GEVYAECVSDSSIFVQ     319
            : *     . **** *.  ::.  :. .* :** *: *     . *:.   ..  :*::

SMAD1      SRNCNYHHGFHPTT-VCKIPSGCSLKIFNNQEFAQLLAQSV-------------------     396
SMAD2      SPNCNQRYGWHPAT-VCKIPPGCNLKIFNNQEFAALLAQSV-------------------     398
SMAD3      SPNCNQRYGWHPAT-VCKIPPGCNLKIFNNQEFAALLAQSV-------------------     324
SMAD4      SYYLDREAGRAPGDAVHKIYPSAYIKVFDLRQCHRQMQQQAATAQAAAAAQAAAVAGNIP     469
SMAD5      GFTDPSNNKSRFCLGLLSNVNRNSTIENTRRHIGKGVHLYYVG-GEVYAECLSDSSIFVQ     356
SMAD6      SPTLDAPGGRA--LVVRKVPPGYSIKVFDFERSGLLQHAD--------------------     452
SMAD7      SATLDNPDSRT--LLVHKVFPGFSIKAFDYEKAYSLQRPND-------------------     381
SMAD8      SRNCNYQHGFHPAT-VCKIPSGCSLKVFNNQLFAQLLAQSV-------------------     359
           *   :   .      * *:  .  :* *: .        .

SMAD1      ---------------NHGFETVYELTKMCTIRMSFVKGWGAEYHRQDVTSTPCWIEIHLH     441
SMAD2      ---------------NQGFEAVYQLTRMCTIRMSFVKGWGAEYRRQTVTSTPCWIELHLN     443
SMAD3      ---------------NQGFEAVYQLTRMCTIRMSFVKGWGAEYRRQTVTSTPCWIELHLN     369
SMAD4      GPGSVGGIAPAISLSAAAGIGVDDLRRLCILRMSFVKGWGPDYPRQSIKETPCWIEIHLH     529
SMAD5      ---------------NHGFEAVYELTKMCTIRMSFVKGWGAEYHRQDVTSTPCWIEIHLH     441
SMAD6      --------------------AAHGPYDPHSVRISFAKGWGPCYSRQFITSCPCWLEILLN     492
SMAD7      ------------------HEFMQQPWTGFTVQISFVKGWGQCYTRQFISSCPCWLEVIFN     423
SMAD8      ---------------HHGFEVVYELTKMCTIRMSFVKGWGAEYHRQDVTSTPCWIEIHLH     404
                                         :::**.****  * ** :.. ***:*: ::

SMAD1      GPLQWLDKVLTQMGSPHNPISSVS   465
SMAD2      GPLQWLDKVLTQMGSPSVRCSSMS   467
SMAD3      GPLQWLDKVLTQMGSPSIRCSSVS   393
SMAD4      RALQLLDEVLHTMPIADPQPLD--   551
SMAD5      GPLQWLDKVLTQMGSPLNPISSVS   465
SMAD6      NHR---------------------   495
SMAD7      SR----------------------   425
SMAD8      GPLQWLDKVLTQMGSPHNPISSVS   428
```
